# Les séquelles de brûlures cervicales: aspects épidémiologique, clinique et thérapeutique au Maroc

**DOI:** 10.11604/pamj.2015.20.413.6605

**Published:** 2015-04-27

**Authors:** Amine Rafik, Hakim Chabak, Mounia Diouri, Naïma Bahechar, Abdessamad Chlihi

**Affiliations:** 1 Centre National des Brûlés et de Chirurgie Plastique, Casablanca, Maroc

**Keywords:** Brölure, séquelle de brölure, épidémiologie, cou, Burns, sequelae of burns, epidemiology, neck

## Abstract

Les séquelles de brûlures cervicales représentent une entité fréquente des séquelles de brûlure, elles affectent la fonction, l'esthétique et l’état psychologique des patients et peuvent être de traitement difficile. Il s'agit d'une étude rétrospective étalée sur 5 ans de Mars 2009 au Octobre2014, réalisée au centre national des brûlés et de chirurgie plastique au CHU Ibn Rochd Casablanca. Nous avons analysé les caractéristiques épidémiologiqueset cliniques ainsi que les indications et les résultats thérapeutiques chez 300 patients présentant des rétractions cervicales post-brûlure, suivis dans notre formation. Les jeunes femmes étaient le plus souvent touchées (56%). la brûlure thermique par flamme de butane dans le cadre d'accident domestique était l’étiologie la plus fréquente (91%).75% des patients ont été pris en charge dans un délai de 18 mois après avoir présenté une incapacité fonctionnelle. Les brides cervicales modérées et sévères sont les plus fréquentes et représentent respectivement 60% et 16% des cas. Le traitement chirurgical a fait appel aux greffes cutanées dans 67%des cas, aux plasties locales dans 24%des cas et aux lambeaux dans 24% des cas, les résultats sont jugés bons dans 75%des cas et moyens dans 18% des cas, tandis que les cas restants (7%) ont nécessité une reprise chirurgicale. Le traitement des brides cervicales doit être associé à un programme de rééducation adapté, afin d'assurer la pérennité des résultats fonctionnels et esthétiques.

## Introduction

Les brûlures cervicales sont sources de séquelles invalidantes génératrices de préjudices esthétiques et fonctionnels [1]. La précocité et la qualité de la prise en charge initiale sont déterminantes quant aux suites évolutives, se faisant habituellement au stade de maturation cicatricielle et de stabilisation cutanée, la réparation chirurgicale doit être précoce chez l'enfant vu le retentissement sur la croissance surtout au niveau du secteur osseux mandibulaire [2]. Seule une stratégie thérapeutique collégiale peut garantir un résultat de qualité. Le but de notre travail est d'illustrer l'expérience de notre équipe en matière de prise en charge des séquelles de brulures cervicales tout en décrivant leur profil épidémiologique, leurs particularités cliniques ainsi que notre approche thérapeutique et les différentes techniques de réparation adoptées.

## Méthodes

Nous présentons une étude rétrospective descriptive monocentré, étalée sur une période de 5 ans: Mars 2009- Octobre2014 concernant une série de 300 patients menée au centre national des Brûlés et de la Chirurgie Réparatrice, CHU Ibn-Rochd Casablanca. Pour chaque patient, nous avons noté l’âge, le sexe, et les antécédents médicaux et chirurgicaux…. Un examen complet et général a été effectué chez tous nos patients. Les résultats, selon la technique utilisée, le siège et l′étendue de la rétraction ont été évalués par des critères objectifs fonctionnels et esthétiques. En évaluant les résultats post-opératoires, nous avions à l′esprit les critères suivants: résultats esthétiques excellents: cicatrice invisible et aspect normal du cou; résultats esthétiques acceptables: cicatrice visible de près (20 cm), l′aspect anatomique moins bien défini; résultats esthétiques moyens: changement morphologique sans trouble fonctionnel important.

› Résultats esthétiques mauvais: changement morphologique avec un retentissement fonctionnel.

## Résultats

**Figure 1:**
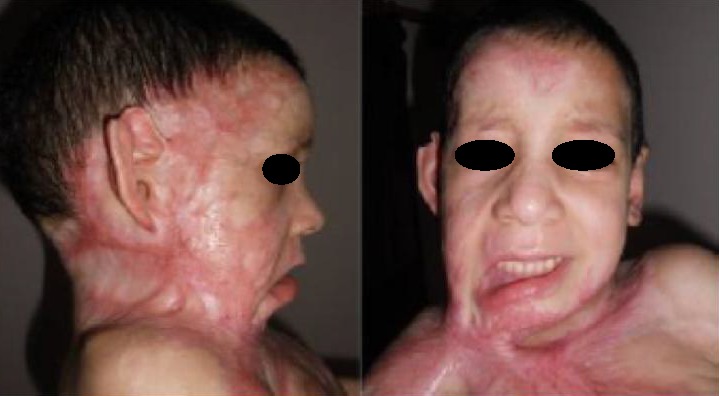
Les étiologies de la brûlure dans notre série

**Figure 2:**
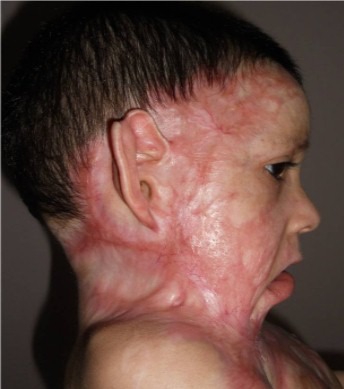
Le délai entre la consultation et l'apparition des séquelles de brûlures cervicales

**Figure 3:**
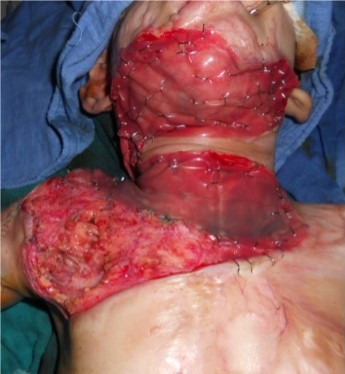
Patiente de 3ans présente une forme spectaculaire d'une symphyse sterno-mentonnière, accompagnée d'une attraction majeure d'origine thoracique, avec une rétraction axillaire sévère et une atteinte du tiers inférieur de la face, on note aussi un ectropion palpébral, et une éversion de labiale inférieure.

**Figure 4:**
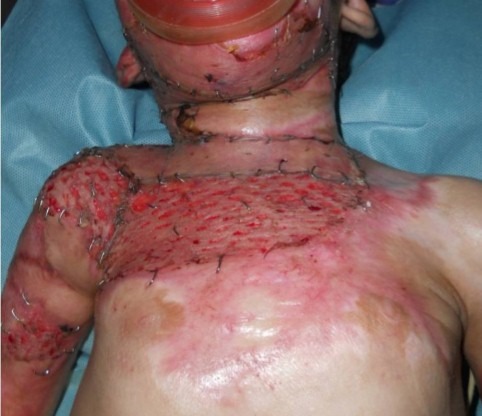
Les techniques chirurgicales utilisées chez nos patients

La population jeune reste la plus touchée avec une légère prédominance féminine de 56%. Les accidents domestiques viennent au premier rang dans 87% des cas et occasionnent une Brûlure thermique dans 91% des cas (Figure 1). Le délai de consultation et de prise en charge reste variable et relativement long (Figure 2). Les lésions cervicales rencontrées se répartissent selon la classification d'Achauer [3] (Tableau 1). Les formes historiques de symphyse sterno- mentonnière sont devenues rares dans notre contexte. Elles étaient rencontrées dans Quinze cas ayant échappé à notre prise en charge initiale et ayant consulté à un stade séquellaire avancée (Figure 3). Nos indications chirurgicales se dégageaient essentiellement de la topographie des brides rétractiles et des pertes de substances consécutives à l'excision des placards cicatriciels. Le traitement conventionnel, les greffes cutanées secondaires ainsi que la physiothérapie sont les piliers majeurs de notre prise en charge initiale et de notre volet thérapeutique préventif, Le traitement chirurgical s'est avéré délicat du fait du risque élevé d'intubation difficile, Parmi les 300 patients 18% ont été d'intubation difficile. Le traitement chirurgical a fait appel essentiellement aux procèdes résumés dans la Figure 4. Les procédés de transferts libres n'ont pas été utilisés dans cette série.

**Tableau 1:** Classification des séquelles de brûlure cervicales selon B.M.ACHAUER [3]

Atteinte	Nombre (N)	Pourcentage (%)
Bride cicatriciel légère	73	24
Placards cicatriciel modéré	179	60
Placards cicatriciel sévère	48	16

## Discussion

La région cervico-faciale est une zone découverte, les séquelles physique et esthétique engendrées par la brûlure affectent psychologiquement les patients, surtout jeunes, et peuvent conduire à une dépression grave et une altération de qualité de vie. Dans notre série, un total de 300 patients avec des séquelles de brûlure cervicale ont été gérés dans cette étude. 35,5% de nos patients étaient dans la tranche d′âge des 24-32 ans avec un âge moyen de 25ans et une légère prédominance féminine. Ces résultats sont similaires à ceux retrouvés dans la littérature [1, 4, 5] (Tableau 2). Les brides cervicales, chez 75.3% de nos patients, ont été causées par des brûlures thermiques, en l'occurrence la flamme de butane (bouteille de gaz de 3 kg). Ce type de brûlures, est très fréquent dans notre contexte et ce produit habituellement au cours d'une manipulation domestique imprudente. Ahuja [6] a observé que dans les pays en voie de développement, comme l′Inde, les brûlures thermiques sont les plus fréquentes en comparaison des brûlures électriques ou chimiques qui sont plus rares. Il ajoute que la plupart de ces brûlures sont accidentelles et se produisent dans la cuisine, suggérant que le mode de vie domestique a une incidence importante sur le phénomène. Dans cette étude, les rétractions modérées, sévères et étendues, sont présentes chez 76% des patients. Wilson [7] a remarqué que les brides sévères sont plus fréquemment observées dans les pays en voie de développement, résultat à la fois de l′utilisation généralisée des feux à foyers ouverts et de l′insuffisance des soins des brulures dans ces pays. Par ailleurs, 63,6% de nos patients ont été opérés moins de 18 mois après la brûlure. Les indications de libération dans notre étude étaient les limitations fonctionnelles en plus de la défiguration. C. Angrigiani [8] a traité 86 patients dont l′intervalle entre la brûlure et la reconstruction a été de 6 mois à 3 ans. Dans notre série, les brides cicatricielles étroites ont été excisées avec des plasties locales, alors que la libération a été principalement utilisée dans les atteintes sévères et étendues. Une exclusion du muscle peaucier, selon Texier [9], permet d′obtenir une meilleure hyper-extension de la tête et un angle cervico-mentonnier mieux dessiné. Bhattacharya [10] a excisé tout le placard cicatriciel du menton au manubrium. Zhang, Y. X. et al [11] ont réalisés une platysmaplastie après la libération des brides sévères pour prévenir la récidive, et approfondir l′angle cervicomental.

**Tableau 2:** Revue de la littérature sur les séquelles de brulure cervicales

	N. B Mody [1] (n = 22)	Tsai F.C [4] (n = 28)	M. Moustafa [5] (n = 264)	Notre étude (n = 300)
Age moyen	28 ans	41ans	23 ans	25ans
Sexe	F = 77.3%	F = 17.5%	F = 66.3%	F = 56%
Etiologies	Thermique 81.8%	Thermique 85.7%	Thermique + + +	Thermique 91%
Classification d'Achauer's	Modéré et sévère + + + (77.3%)	-	Modéré et sévère + + + 83.7%	Modéré et sévère + + + 76%

Au cours de cette étude, on a eu recours à la greffe cutanée sans intégra chez 180 patients, soit 89.5% des cas avec prélèvement de la peau semi-épaisse au niveau de la cuisse, la jambe ou l′abdomen. Il y a eu récidive de la rétraction chez quinze de nos patients (7.6%) exigeant une reprise chirurgicale. Waymach et al [12] a rapporté un taux de récidive de 17%, après la greffe cutanée et l′utilisation d′une attelle d'hyper-extension du cou pendant plus d’ 1 an. Vingt-un patients ont bénéficié d'une greffe d'intégra^®^ suivie d'une greffe de peau mince; les résultats esthétiques et fonctionnels ont été jugés satisfaisants. L'intérêt de l'apport d'un substitut de derme Intégra^®^ est de réduire la récidive, ce procédé a été évalué par plusieurs études multicentriques [13–15]. Cependant, la seule limite d'utilisation du derme artificiel est son coût relativement élevé. Les lambeaux ont été utilisés pour couvrir les pertes de substance résultantes chez 29 patients dans notre étude. Ils offrent un potentiel de croissance chez l'enfant, et accompagnent la récupération des amplitudes articulaires chez l'adulte après la rééducation. B.G.H. Lambarty [16] a utilisé le lambeau supra-claviculaire, qu′il a décrit, chez une patiente de 19 ans qui présentait un placard cicatriciel cervical sévère. Norbert Pallua [17] l'a modifié et l'a utilisé pour libérer les contractures mentosternales post-brulures chez 14 patients. Nous avons utilisé le lambeau supra-claviculaire chez six de nos patients. 93% de nos patients avaient une fonction et un aspect esthétique du cou acceptables après 18 mois de rééducation. Le déficit fonctionnel et le gêne esthétique sont aussi des critères utilisés par Nath [18] pour déterminer les résultats post-opératoires et l’évolution cicatricielle.

## Conclusion

La prévention primaire est fondamentale pour réduire l'incidence de cette affection elle passe avant tout par L’éducation et la sensibilisation des populations. La qualité du résultat repose sur une prise en charge globale, médico-chirurgicale. Les approches thérapeutiques chirurgicales les plus adaptées sont celles qui cherchent à améliorer le résultat aussi bien fonctionnel qu'esthétique.
